# The Extent to Which Animal Forms Undergo Change

**Published:** 1883-04

**Authors:** C. M. O’Leary


### Art. IX.—THE EXTENT TO WHICH ANIMAL FORMS UNDERGO CHANGE.

BY C. M. O'LEARY, M.D., L.L.D.

IT has lately, and with apparently good reason, become the fashion for magazines, instead of confining themselves to the exposition of a distinct set of views, to devote themselves to the dissemination of diverse and even opposite notions touching similar matters in the hope that from the clash of opinions the spark of truth may be struck. It is in this spirit that the writer has turned his attention to an article from the pen of Dr. Hubbard W. Mitchell, which appeared in the January number of the Journal. The article in question possesses the undeniable merit of lucidity, and the reader who is anxious to obtain a rapid and brief summary of the most advanced stage of the doctrine of evolution may find it here. I consider, however, that Dr. Mitchell has adopted some extreme opinions without having thoroughly sifted the arguments on which they rest or considered the consequences to which they lead. I will mention two instances in which this seems to me to have occurred. Considering the aptitude which certain atoms possess to combine with others, and the number of specifically different substances that thence arise, the Doctor has formulated a theory to the following effect: If $C^4$. $H^5$. O. combine to form ether, which, by the addition of H. O., is converted into alcohol, ($C^4$. $H^6$. $O^2$.) why should not these same elements separately and in the same proportions, or united and in different proportions, or separately and in different proportions, go to form the basis of organic life? If an analysis of organized substances gives as a result elementary bodies through the agency of chemical force why should not this same force effect a synthesis of the same elements and give us organized substances endowed with vitality. Very briefly stated this is the theory of Dr. Mitchell. He maintains not only the possible but the actual transition of non-living inorganic substances to living and organic substances through the operation of chemical force. I would in the first place remark that Dr. Mitchell does not adduce a single argument in support of his position and yet he holds to it as a theory advanced by himself. Logic requires that a hypothesis be supported at least by the necessity of the facts which it is invoked by its author to explain, yet such necessity is not even adverted to here. In the next place I contend that the facts all point in the opposite direction and that the so-called spontaneous origin of organic life is a hypothesis plainly at variance with the results of experience and observation. Not only has chemistry failed to produce a living organism from any and every conceivable combination of elementary substances, but the records of biology constantly prove the truth of the dictum *omne vivum ex vivo.* And reason corroborates the statement, for it is plain that we destroy or eliminate a form called life when we analyse, and that in synthesis we only combine dead forms which can give in the aggregate nothing which they did not contain either potentially or actually in their elementary condition *nemo dat quod non habet.* Now we see nothing resembling vital force in chemical combinations, while we can account for all their varied phenomena by a reference to the elements from which they sprang. The doctrine of abiogenesis is generally rejected to-day, especially since Prof. Tyndall's experiments have proved the impossibility of the transition of inorganic matter into vitalized organic substance, experiments which led him to the abandonment of the theory of spontaneous generation. More lately the interesting experiments conducted by Pasteur, Schwann and Schroeder, and which have led to the marvellous innovation in surgery, known as "Listerism," prove convincingly that all living organisms must spring from a germ either actually or potentially endowed with vitality. These experimenters have been untiring in their efforts to show that a germ alone, itself the product of vital action, can develop life, and their conclusions have led to practical results of the utmost value.

Schwann has proved that after the fertilizing powers of organic substances had been removed they remained unaffected by pure oxygen devoid of vitalized germs. The theory that organic life was due to gases commingled with the air succumbed to the facts established by these investigators. Thus organic fluids which had been filtered through cottonwood by Schroeder and Dusch were preserved for an indefinite period of time in the presence of heated air, *i. e.*, air from which vital germs had been excluded. With regard to wounds, the labors of Guerin have proved that it was useless to exclude merely the air since it *per se* had no effect upon putrefaction, but that something in the air, organized and endowed with a potential vitality, had to be excluded. The abiogenetic theory dates back to Aristotle, who held precisely the same views as Dr. Mitchell, though not couched in the same scientific formulæ. Harvey adopted the opinion of Aristotle and held that "equivocal generation" or production of living substances out of non-living matter can alone account for the existence and operation of vital force. We know that the immense majority of plants and animals take their origin in actually vital germs and the whole course of modern inquiry tends to the establishment of the law that out of chemical elements alone no living substance can proceed. The facts of fermentation especially point to this conclusion. The *Schizomycetes*, improperly called *Bacteria*, are the micro organism to whose presence fermentation is due. If by artificial means we exclude this germinating element fermentation either does not take place or is arrested. These multiply according to the special law governing the class of cells to which they belong. As they multiply they require new pabulum, which they readily obtain if deposited in a suitable medium, but as speedily die out when not thus congenially situated. We thus have alcoholic, butyric, lactic and putrefactive fermentation which never can take place in the mere presence of the chemical components ofthe *Schizomycetes*, but requires for its inception and continuance the presence of the vital germ itself. Dr. Mitchell, it is true, does not assert that this process of evolution from inorganic elements into living tissues takes place now, but he endeavors to account for the existence of the first vitalized cell in this manner. Time, however, is an accidental circumstance and vitality an essential one, and certainly what cannot take place now could not have taken place myriads of centuries ago without a breach of the laws to which the physical universe is subject. Once this mysterious life force admitted how powerful becomes its operation! Chemical elements are readily appropriated and assimulated by it and made to share its vitalizing influence. It is the alembic wherein the crude materials out of which the universe is built, are brought together, duly commingled and lifted up to the high plane of living organic structures. It is the breath that vivifies inert masses and can no more be the outcome of organic elements than heat and light can emanate from the chaos of dead worlds. It is not only an assumption therefore to state that the protoplasm of cell life is the result of the agency of chemical forces, but the statement is so far adverse to the facts and opposed to the drift of scientific research.

I come now to the second point wherein it appears to me Dr. Mitchell has transcended the domain of facts. It is true he has the authority of Darwin and Wallace together with the whole school of advanced evolutionists to sustain him in the position he has taken, but there are many grave authors who will not subscribe to the doctrine of a primordial pangenetic protoplasm. Dr. Mitchell writes: "If our theory is correct, that the cell acquired its vitality or life through chemical force, the first step is made plain and we can now go on and follow the law of evolution from the first dawn of life upon the earth through all its manifold and successive changes, tracing step by step living forms as they proceed to a higher and still higher form until we reach that level upon which we ourselves stand in the full enjoyment of a completely developed organization, animated and controlled by a perfect brain." I have quoted the Doctor's words at length in order that the strictures I am about to make may be seen to have a direct reference to his views.

According to Dr. Mitchell all living structures had their origin in a homogeneous protoplasm which became organized and specialized through heterogeneity. The law of natural selection is invoked to sustain this view and the simplicity and efficiency of the law in many interesting cases lends color to its supposed adequacy to account for the transmutation of species. Now the law of natural selection is not competent to explain all the facts for the explanation of which it is invoked and notably, this is the case with reference to many incipient structures, as has been proved by St. George Mivart in his treatise "On the Genesis of Species." If the long neck of the giraffe were the result of necessity, why should not the other ungulata of South Africa have been similarly favored as having been exposed to similar conditions? Yet when the giraffe feasted on the foliage of tall trees its congeners perished by hundreds for want of a suitable surface vegetation. Here the law of selection is greviously at fault and we must seek elsewhere for the structural peculiarity of this animal than in the doctrine of progressive variation. Like difficulties present themselves when we study the remarkable phenomena of mimicry, or look at the heads of certain flat fishes and consider the origin and constancy of the vertebrate limbs. The more deeply we ponder over these facts the more apparent becomes the inadequacy of Darwin's theory to account for the preservation and intensification of incipient specific and generic characters. And yet it is only through the application of the law of natural selection to structures presenting such characters that we can admit the evolution of animal forms from a lower to a higher type. Moreover such progressive evolution is opposed to the stability and reality of species and leads to that most unscientific doctrine the transmutation of species. M. A. De Quatrefages, than who there the is no higher authority in zoölogy, has thoroughly established fact of the reality of species, and we have but to reflect a moment over the peculiar characters of hybridism and mongrelism to became convinced that species is not an artificial and conventional grouping. Mere morphological peculiarities, however marked, are not sufficient to constitute an essential difference between individuals. We must turn our attention, moreover to the facts of physiology and filiation. It is true that more notable differences in point of form prevail between nearly correleated individuals than between those which profound differences separate. Among dogs and pigeons this is evident at a glance and yet the closeness of specific connection between any and all individuals of these two families is established by the fertility of their mongrel breed. The greyhound and the pug, however, morphologically distinct give birth to an offspring even more fruitful than the parents and thereby prove their physiolgical unity. How different the case is with the horse and the ass, which present numerous points of close morphological similarity. The mule was known to the Israelites in the very earliest times as well as to the Greeks of the Homeric period and yet he remains sterile and unchanged up to the present time. Now if there existed a change of type in specific characters, surely such tendency would have manifested itself in this case long before now. The *Titires* and *Musmons*, the products respectively of crossings between the he-goat and the sheep and the ram with the she-goat were so designated by the Romans and continue to exhibit the same immutability to the present day. Buffon and Daubenson often strove to reproduce titires and musmons, but their efforts were unavailing and we may hence logically conclude that these animals are crystalized in their characters and incapable of change.

The chief difficulty in the way of the acceptance of organic evolution, as predicted by Darwin and Wallace, arises from the undue importance it attaches to morphological characters, and its consequent neglect of the value of physiological and ancestral differences. It is no wonder that, attributing everything to form, these biologists could see in specific marks mere footsteps in sand, shifting and variable differences that mean nothing. On the other hand a due consideration of physiological peculiarities necessarily leads to the determination of an essential difference between species and species.

The closer morphological similarities are accompanied by permanent physiological differences the more apparent must become the impossibility of specific transmutation. The tailed catarrhine apes, which Darwin holds to be our first direct ancestors, still exist with all the characteristics of which according to Darwin, they were possessed at the time when an upper departure from type occurred. Now the pertinent query suggests itself, why did only a certain number undergo this progressive metamorphosis, and why did the others so far from merging into other types retain their essential characters up to the present time amidst environments that are radically different? Haeckel is plunged in the same embarassment, and in endeavoring to trace human origin from his so-called *Monera* through twenty-one progressive stages of evolution he finds himself confronted by insuperable difficulties. Having traced to his satisfaction the series of developments to the twentieth stage he is utterly unable to determine the nature of the change from the Chimpanzee to man, and so he imagines a purely mythical being which he calls the pithecoid man, or ape man, to which he denies the gift of articulate speech together with human intelligence and self-consciousness.

For additional objections to Darwin's and Haeckel's theories of transmutation the reader is referred to the chapter on the "Theories of Darwin and Haeckel," in Quatrefrage's treatise on the Human Species, and to the chapter on "Specific Stability," in St. George Mivart's work on "The Genesis of Species." Space forbids me from entering into the matter more fully, but I think I have hinted at enough to make it manifest that we cannot adopt the theory of an evolution which disregards the permanence and reality of species without more impressive arguments than have hitherto been advanced. Few writers have dressed out the views of Darwin in more attractive garb than Dr. Mitchell, and the plausible assurance with which he intimates that there can be no difficulty in the way of their acceptance being calculated to win over many an unwary reader has led me to pen the above objections to them.

